# Outcome reporting recommendations for clinical trial protocols and reports: a scoping review

**DOI:** 10.1186/s13063-020-04440-w

**Published:** 2020-07-08

**Authors:** Nancy J. Butcher, Emma J. Mew, Andrea Monsour, An-Wen Chan, David Moher, Martin Offringa

**Affiliations:** 1 Child Health Evaluative Sciences, The Hospital for Sick Children Research Institute, Peter Gilgan Centre for Research and Learning, Toronto, ON Canada; 2 Department of Medicine, Women’s College Research Institute, University of Toronto, Toronto, ON Canada; 3 Centre for Journalology, Clinical Epidemiology Program, Ottawa Hospital Research Institute, Ottawa, ON Canada; 4 School of Epidemiology and Public Health, University of Ottawa, Ottawa, ON Canada; 5 Institute of Health Policy, Management and Evaluation, University of Toronto, Toronto, ON Canada; 6 Division of Neonatology, The Hospital for Sick Children, Toronto, ON Canada

**Keywords:** Trial, Trial protocols, Outcome, Endpoint, Reporting guideline, SPIRIT, CONSORT

## Abstract

**Background:**

Clinicians, patients, and policy-makers rely on published evidence from clinical trials to help inform decision-making. A lack of complete and transparent reporting of the investigated trial outcomes limits reproducibility of results and knowledge synthesis efforts, and contributes to outcome switching and other reporting biases. Outcome-specific extensions for the Standard Protocol Items: Recommendations for Interventional Trials (SPIRIT-Outcomes) and Consolidated Standards of Reporting Trials (CONSORT-Outcomes) reporting guidelines are under development to facilitate harmonized reporting of outcomes in trial protocols and reports. The aim of this review was to identify and synthesize existing guidance for trial outcome reporting to inform extension development.

**Methods:**

We searched for documents published in the last 10 years that provided guidance on trial outcome reporting using: an electronic bibliographic database search (MEDLINE and the Cochrane Methodology Register); a grey literature search; and solicitation of colleagues using a snowballing approach. Two reviewers completed title and abstract screening, full-text screening, and data charting after training. Extracted trial outcome reporting guidance was compared with candidate reporting items to support, refute, or refine the items and to assess the need for the development of additional items.

**Results:**

In total, 1758 trial outcome reporting recommendations were identified within 244 eligible documents. The majority of documents were published by academic journals (72%). Comparison of each recommendation with the initial list of 70 candidate items led to the development of an additional 62 items, producing 132 candidate items. The items encompassed outcome selection, definition, measurement, analysis, interpretation, and reporting of modifications between trial documents. The total number of documents supporting each candidate item ranged widely (median 5, range 0–84 documents per item), illustrating heterogeneity in the recommendations currently available for outcome reporting across a large and diverse sample of sources.

**Conclusions:**

Outcome reporting guidance for clinical trial protocols and reports lacks consistency and is spread across a large number of sources that may be challenging to access and implement in practice. Evidence and consensus-based guidance, currently in development (SPIRIT-Outcomes and CONSORT-Outcomes), may help authors adequately describe trial outcomes in protocols and reports transparently and completely to help reduce avoidable research waste.

## Background

Clinical trials, when appropriately designed, conducted, and reported, are a gold-standard study design for generating primary evidence on treatment efficacy, effectiveness, and safety. In clinical trials, outcomes (sometimes referred to as endpoints or outcome measures) are measured to examine the effect of the intervention on trial participants. The findings of the trial thus rest critically on the trial outcomes. As data accumulate across different clinical trials for specific interventions and outcomes, the outcome data published in clinical trial reports are ideally synthesized through systematic reviews and meta-analyses into a single estimate of effect that can inform clinical and policy-making decisions. This evidence generation and knowledge synthesis process enables the practice of evidence-based medicine. This process is facilitated by the complete and prospective definition of trial outcomes. Appropriate outcome selection and description are important for obtaining ethical and regulatory approvals, ensuring the trial team conducts the trial consistently and, ultimately, provides transparency of methods and facilitates the interpretation of the trial results.

Despite the importance of trial outcomes, it is well established in the biomedical literature that key information about how trial outcomes were selected, defined, measured, and analysed is often missing or poorly reported across trial documents and information sources [1–8]. A lack of complete and transparent reporting of trial outcomes limits critical appraisal, reproducibility of results, and knowledge synthesis efforts, and enables the introduction of bias into the published literature by leaving room for outcome switching and selective reporting. There is evidence that up to 60% of trials change, omit, or introduce a new primary outcome between the planned trial protocol and the published trial report [3, 9–12]. Secondary outcomes have been less studied, but may be even more prone to bias and inadequate reporting [12, 13]. Deficient outcome reporting, either through selective reporting of the measured outcomes or incompletely pre-specifying and defining essential components of the reported outcome, facilitates undetectable data “cherry-picking” in the primary reports and has the potential to impact the conclusions of systematic reviews and meta-analyses [14, 15].

Although there is an established need among the scientific community to improve the reporting of trial outcomes [5, 16–19], it remains unknown what actually constitutes useful, complete reporting of trial outcomes to knowledge users. The well-established Standard Protocol Items: Recommendations for Interventional Trials (SPIRIT) [20] and Consolidated Standards of Reporting Trials (CONSORT) [21] reporting guidelines provide guidance on what to include in clinical trial protocols and reports, respectively. Yet although SPIRIT and CONSORT provide general guidance on how to report trial outcomes [20, 21], and have been extended to cover patient-reported outcomes [22, 23] and harms [24], there remains no standard evidence-based guidance that is applicable to all outcome types, disease areas, and populations for trial protocols and published reports.

An international group of experts and knowledge users [25] has therefore convened to develop outcome-specific reporting extensions for the SPIRIT and CONSORT reporting guidelines. Originally referred to as the SPIRIT-InsPECT and CONSORT-InsPECT (Instrument for reporting Planned Endpoints in Clinical Trials) reporting extensions, the final products will be referred to as the SPIRIT-Outcomes and CONSORT-Outcomes extensions in response to stakeholder and end-user input. These extensions will be complementary to the work of the Core Outcome Measures in Effectiveness Trials (COMET) Initiative and core outcome sets; core outcome sets standardize which outcomes should be measured for particular health conditions, whereas SPIRIT-Outcomes and CONSORT-Outcomes will provide standard harmonized guidance on how outcomes should be reported [26].

The SPIRIT-Outcomes and CONSORT-Outcomes extensions are being developed in accordance with the methodological framework created by members of the Enhancing Quality and Transparency of Health Research Quality (EQUATOR) Network for reporting guideline development, including a literature review to identify and synthesize existing reporting guidance [27]. The protocol to develop these guidelines has been published previously [28]. An initial list of 70 candidate trial outcome reporting items was first developed through an environmental scan of academic and regulatory publications, and consultations with methodologists and knowledge users including clinicians, guideline developers, and trialists [28–30]. These 70 items were organized into ten descriptive categories: What: description of the outcome; Why: rationale for selecting the outcome; How: the way the outcome is measured; Who: source of information of the outcome; Where: assessment location and setting of the outcome; When: timing of measurement of the outcome; Outcome data management and analyses; Missing outcome data; Interpretation; and Modifications.

The purpose of this scoping review was to identify and synthesize existing guidance for outcome reporting in clinical trials and protocols to inform the development of the SPIRIT-Outcomes and CONSORT-Outcomes extensions. The results of this scoping review were presented during the web-based Delphi study and the in-person consensus meeting. A scoping review approach, which is a form of knowledge synthesis used to map concepts, sources, and evidence underpinning a research area [31, 32], was selected given the purpose of this review. The specific research questions that this review sought to address were: what published guidance exists on the reporting of outcomes for clinical trial protocols and reports; does the identified guidance support or refute each candidate item as a reporting item for clinical trial protocols or reports; and does any identified guidance support the creation of additional candidate items or the refinement of existing candidate items?

## Methods

This review was prepared in accordance with the PRISMA extension for Scoping Reviews reporting guideline (see Additional File 1: eTable 1) [33]. The protocol for this review has been published elsewhere [30, 34]. This scoping review did not require ethics approval from our institution.

### Eligibility criteria

Documents that provided guidance (advice or formal recommendation) or a checklist describing outcome-specific information that should be included in a clinical trial protocol or report were eligible if published in the last 10 years in a language that our team could read (English, French, or Dutch). Dates were restricted to the last 10 years from the time of review commencement to focus the review to inform the update and extension of existing guidance provided by CONSORT (published in 2010) and SPIRIT (published in 2013) on outcome reporting and to increase feasibility related to the large number of documents identified in our preliminary searches. There were no restrictions on population, trial design, or outcome type. We only included documents that provided explicit guidance (“stated clearly and in detail, leaving no room for confusion or doubt” [35], such that the guidance must specifically state that the information should be included in a clinical trial protocol or report) [36]. An example of included guidance follows from the CONSORT-PRO extension: “Evidence of patient-reported outcome instrument validity and reliability should be provided or cited, if available” [36].

### Information sources

Documents were searched for using: an electronic bibliographic database search (MEDLINE and the Cochrane Methodology Register; see eTable 2 in Additional file 2 for search strategy), developed in close consultation with an experienced research librarian, and searched from inception to 19 March 2018; a grey literature search; solicitation of colleagues; and reference list searching. Eligible document types included review articles, reporting guidelines, recommendation/guidance documents, commentary/opinion pieces/letters, regulatory documents, government reports, ethics review board documents, websites, funder documents, and other trial-related documents such as trial protocol templates.

The grey literature search methods included a systematic search of Google (www.google.com) using 40 combinations of key words (e.g., “trial outcome guidance”, “trial protocol outcome recommendations”; see eTable 3 in Additional file 3 for a complete list). The first five pages of the search results for each key term were reviewed (10 hits per page, leading to 2000 Google hits screened in total). Documents were also searched for using a targeted website search of 41 relevant websites (e.g., the EQUATOR Network, Health Canada, the Agency for Healthcare Research and Quality; see eTable 3 in Additional file 3) identified by the review team, solicitation of colleagues, and use of a tool for searching health-related grey literature [37]. Website searching included screening of the homepage and relevant subpages of each website. When applicable, the term “outcome” and its synonyms were searched for using the internal search feature of the website. We searched online for forms and guidelines from an international sample of ethics review boards, as ethics boards are responsible for evaluating proposed trials including the selection, measurement, and analyses of trial outcomes. We restricted the ethics review board search to five major research universities and five major research hospitals (considered likely to be experienced in reviewing and providing guidance on clinical trials) in four English-speaking countries: United States, United Kingdom, Canada, and Australia (see eTable 3 in Additional file 3). This approach helped to limit the search to a manageable sample of international ethics review board guidance. To ensure diverse geographic representation of documents from ethics review boards, as some countries yielded substantially more documents than others, documents were randomly selected from each of the four selected countries (i.e., 25% of documents were from each country), amounting to approximately half of the number of the total ethics review board documents initially identified.

Additional documents and sources from experts were obtained by contacting all founding members of the “InsPECT Group” [25]. This included 18 trialists, methodologists, knowledge synthesis experts, clinicians, and reporting guideline developers from around the world [28]. We asked each expert to identify documents, relevant websites, ethics review boards, and additional experts who may have further information. All recommended experts were contacted with the same request. Given the comprehensiveness of our search strategies and the large number of documents identified as eligible for inclusion, we performed reference list searching only for included documents identified via Google searching, as this document set encompassed the diversity of sources and document types eligible for inclusion (e.g., academic publications, websites).

### Selection of sources of evidence

A trained team member (L. Saeed) performed the final electronic bibliographic database searches and exported the search results into EndNote version X8 [38] to remove all duplicates. All other data sources were first de-duplicated within each source manually, and then de-duplicated between already screened sources, leaving only new documents to move forward for “charting” (in scoping reviews, the data extraction process is referred to as charting the results) [32, 33].

#### Initial screening

All screening and data charting forms are available on the Open Science Framework [39]. Titles and abstracts of documents retrieved from the electronic bibliographic database search were screened for potential eligibility by one of two reviewers with graduate-level epidemiological training (AM, EJM) before full texts were thoroughly examined. The two reviewers assessed 90 citations as a practice set and reviewed the results with a senior team member (NJB). The reviewers then screened a randomly selected training set of 100 documents from the electronic bibliographic database search and achieved 93% observed agreement and 71% chance agreement, yielding a Cohen’s κ score of 0.76 (substantial agreement [40]). The remaining search results were then divided and each independently screened by one of the two reviewers, with periodic verification checks performed by NJB. One reviewer (AM) screened and charted all website search results. Documents gathered from the ethics review board searches (by L. Saeed) and from the solicitation of experts moved directly to full-text review and charting by EJM.

#### Full-text screening

The reviewers (AM, EJM) performed full-text screening for eligibility using a similar process as for title and abstract screening. A sample of 35 documents identified from title and abstract screening were assessed for eligibility. The observed agreement rate was 94% (33 of 35 documents). The included documents (*n* = 14) were charted in duplicate, and the reviewers examined their charting results and resolved any discrepancies through discussion. Following review of the agreement results by a senior team member (NJB), the remaining search results were divided and independently screened and charted by one of the two reviewers, with periodic verification checks performed by NJB. Full-text screening and reasons for exclusion were logged using a standardized form [39] developed using Research Electronic Data Capture (REDCap) software [41].

### Data charting process

The included documents proceeded to undergo data charting using a standardized charting form [39] developed using REDCap software [41]. Prior to data charting, 11 documents were piloted through the full-text screening form and the charting form by EJM and AM (AM was not involved in developing the forms), and the forms were modified as necessary following review of the form testing with NJB and MO. The reviewers (AM, EJM) charted data that included information such as characteristics of the document (e.g., publication type, article title, last name of first author, publication year, publisher) as well as the scope and characteristics for each of the specific recommendations extracted from each included document (e.g., whether the recommendation was specific to clinical trial protocols or reports, or specific to type of outcomes, trial design, or population). Given the nature of this review, a risk of bias assessment or formal quality appraisal of included documents was not performed. To help gauge the credibility of recommendations gathered, we categorized the type(s) of recommendation as made with supporting empirical evidence provided within the source document (e.g., based on findings from a literature review or expert consensus methods) and/or citation(s) provided to other documents (e.g., citation provided to an existing reporting guideline), or neither.

### Synthesis of results

Recommendations identified within the included documents were compared with the candidate outcome reporting items to support, refute, or refine item content and to assess the need for the development of additional candidate items. To achieve these aims, the reviewers (AM and EJM) mapped each recommendation gathered to existing candidate items or one of the ten descriptive categories, supported by full-text extraction captured in free text boxes within the charting form. Recommendations that did not fall within the scope of any existing candidate items or categories were captured in free text boxes. Eight in-person meetings were held by members of the “InsPECT Operations Team” [25, 28] over a 2-month period to review these recommendations and to develop any new candidate reporting items or refine existing candidate items to better reflect the concepts/wording in the literature. Attendance was required by the review lead author (NJB), the senior author (MO), and at least three other members of the Operations Team (EJM, AM, L. Saeed, A. Chee-a-tow). After completion of data collection, the mapping results of recommendations to each candidate item were reviewed by NJB in their entirety and finalized by consensus with the two reviewers (EJM, AM). The wording of the candidate items was then clarified as necessary and finalized by the Operations Team. Data analysis included descriptive quantitative measures (counts and frequencies) to characterize the guidance document characteristics and their recommendations.

## Results

**Fig. 1:**
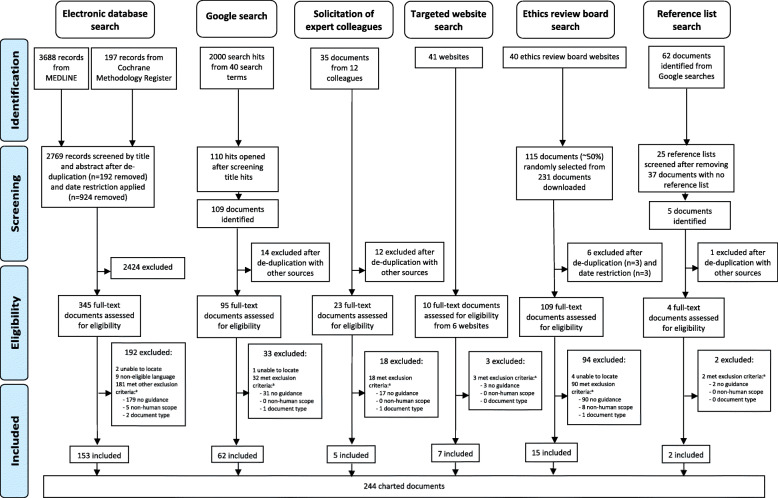
Preferred Reporting Items for Systematic Reviews and Meta-Analyses (PRISMA) flow diagram for the scoping review of documents containing trial outcome reporting recommendations. ^a^These exclusion criteria counts are not mutually exclusive

The full dataset is available on the Open Science Framework [39]. The electronic database literature search yielded 2769 unique references, of which 153 documents were found to be eligible and included (Fig. 1). The Google searches (2000 hits assessed in total) led to the inclusion of 62 documents. An additional seven documents were identified and included from the targeted website search (41 websites assessed). There were five documents included from 12 experts (33 were contacted in total), 15 documents from 40 ethics review boards websites, and two from reference list screening. In total, 244 unique documents were included (Fig. 1).

The majority of the included documents were published by academic journals (72%; Table 1). Other publishers include hospitals, universities, and research organizations as well as governments and non-governmental organizations. All but one document were published in English. The types of documents included varied but were primarily literature reviews (30%), recommendation/guidance documents (24%), commentary/opinion pieces/letters (12%), or reporting guidelines (14%; Table 1).

**Table 1:** General characteristics of the included documents (*n* = 244)

	***N*** (%)
Document publisher	
Academic journal	176 (72.1)
Hospital/university/research organization	31 (12.7)
Government	21 (8.6)
Non-governmental organization	16 (6.6)
Document type	
Literature review	74 (30.3)
Assessment of reporting completeness^a^	39 (16.0)
Systematic/scoping review	28 (11.5)
Other type of review	7 (2.9)
Recommendation/guidance document	59 (24.2)
Commentary/opinion piece/letter	30 (12.3)
Reporting guideline	34 (13.9)
Trial protocol template	16 (6.6)
Research ethics board document	11 (4.5)
Regulatory document	6 (2.5)
Website	5 (2.0)
Government report	3 (1.2)
Other^b^	6 (2.5)
Publication year	
2008–2010	35 (14.3)
2011–2013	69 (28.2)
2014–2016	70 (28.7)
2017–2018^c^	56 (23.0)
Not reported	14 (5.7)
Language	
English	243 (99.6)
French	1 (0.4)
Dutch	0 (0)

^a^Includes any type of literature review that aimed to assess the completeness of reporting in the included articles from either an original review or a secondary analyses of documents included in a prior review

^b^Includes reporting guideline development protocols (*n* = 2) and a reporting guideline pilot study, checklist for peer-reviewers, statistical analysis plan template, and article evaluating an outcome measurement instrument (*n* = 1 each)

^c^Until time of search (19 March 2018)

Of the included documents, 45 (18%) had a primary focus on trial outcome reporting (e.g., the SPIRIT-PRO reporting guideline [22], a journal commentary on selective outcome reporting [42]). Approximately 40% of the documents were focused on specific age group(s) and/or clinical area(s). Of the 18 documents with a focus on a specific age group, most (*n* = 12) were focused on paediatric populations (Table 2). The clinical areas ranged widely (Table 2), with the highest numbers of documents focused on the areas of oncology (*n* = 15), mental health (*n* = 10), and oral and gastroenterology (n = 10). Approximately one-third of all included documents (*n* = 85) came from such discipline-specific documents (Table 2).

**Table 2:** Subject focus of the included documents (*n* = 244)

	***N*** (%)
Scope	
Primary focus on trial outcome reporting	45 (18.4)
Primary focus not on trial outcome reporting	199 (81.6)
Demographic focus^a^	
None stated	148 (60.7)
Age focus explicitly stated^a^	18 (7.4)
Paediatric (birth–18 years old)	12 (4.9)
Neonates and/or infants	10 (4.1)
Children	11 (4.5)
Adolescents	11 (4.5)
Adulthood (19–65 years old)	5 (2.0)
Geriatric (> 65 years old)	2 (0.8)
Clinical area focus explicitly stated^a^	85 (34.8)
Oncology	15 (6.1)
Mental health	10 (4.1)
Oral and gastroenterology	10 (4.1)
Obstetrics and gynaecology	8 (3.3)
Rheumatology	8 (3.3)
Surgery	6 (2.5)
Neurology	5 (2.0)
Pain management	4 (1.6)
Haematology	3 (1.2)
Respiratory	3 (1.2)
Alternative medicine	2 (0.8)
Critical care	2 (0.8)
Dermatology	2 (0.8)
Diabetes	2 (0.8)
Infectious diseases	2 (0.8)
Nutrition	2 (0.8)
Other^b^	10 (4.1)

^a^Not mutually exclusive

^b^Anaesthesiology, cardiovascular and metabolism, endocrinology, nephrology, obesity, ophthalmology, palliative care, physical rehabilitation, radiology, and urology (*n* = 1 each)

There were 1758 trial outcome reporting recommendations identified in total within 244 eligible documents. The median number of unique outcome reporting recommendations per guidance document was 4 (range 1–46). Assessment of the focus of each recommendation (Table 3) showed that most recommendations were specifically focused on clinical trial protocols (43%) and/or reports (44%). Others were focused on outcome reporting in trial documents generally, ethics boards submissions, and clinical trial proposals in grant applications (Table 3). Only 15% of recommendations focused on a specific trial phase and/or design (Table 3), although nearly half (*n* = 836, 47%) focused on a specific outcome classification (e.g., primary, secondary) or type (e.g., patient-reported outcomes or harms; Table 3 and Additional file 4: eTable 4).

**Table 3:** Focus of the outcome reporting recommendations (*n* = 1758) identified within 244 eligible documents

	***N*** (%)
Trial document type^a^	
Trial reports	781 (44.4)
Trial protocols	758 (43.1)
General trial reporting	229 (13.0)
Ethics boards documents for trial submissions	21 (1.2)
Study proposal for a trial in grant application(s)	1 (0.06)
Trial type	
No specific focus explicitly stated	1369 (77.9)
All trials	124 (7.1)
Specific trial focus^a^	265 (15.1)
Phase^a^	102 (5.8)
Pilot/feasibility	33 (1.9)
II	45 (2.6)
III	64 (3.6)
Design^a^	102 (5.8)
*N*-of-1	44 (2.5)
Cluster	16 (0.9)
Non-inferiority	14 (0.8)
Equivalence	11 (0.6)
Within person	10 (0.6)
Parallel	9 (0.5)
Crossover	6 (0.3)
Adaptive	6 (0.3)
Superiority	5 (0.3)
Pragmatic	3 (0.2)
Outcomes	
No specific focus explicitly stated	911 (51.8)
All outcomes	11 (0.6)
Specific outcome focus^a^	836 (47.6)
Outcome classification^a^	469 (26.7)
Primary	458 (26.1)
Secondary	326 (18.5)
“Important”	7 (0.4)
Tertiary/exploratory	6 (0.3)
Outcome type^a^	474 (27.0)
Patient-reported outcome	288 (16.4)
Harm/adverse event	116 (6.6)
Biological marker	41 (2.3)
Efficacy outcome	33 (1.9)
Composite outcome	13 (0.7)
Survival/time-to-event outcome	11 (0.6)
Surrogate outcome	8 (0.5)
Clinician-reported outcome	7 (0.4)
Continuous outcome	4 (0.2)
Binary outcome	3 (0.2)
“Unintended” outcome	1 (0.06)

^a^Not mutually exclusive

Of all the recommendations identified, approximately 40% were not supported by any empirical evidence or citations; the remaining 60% were supported by empirical evidence provided within the document and/or citations to other documents (Table 4). The type of empirical evidence provided was most often generated from literature reviews, and/or through expert consensus methods (Table 4). Supporting citations to other documents were provided for about one-third of all recommendations (Table 4); cited documents included a wide range of sources, although were often existing reporting guidelines or guidance documents such as SPIRIT, CONSORT, and their associated extensions.

**Table 4:** Source of evidence provided to support each outcome reporting recommendation (*n* = 1758) identified within 244 eligible documents

	***N*** (%)
Empirical evidence provided within source document and/or citations provided	1027 (58.4)
No empirical evidence or citations provided	731 (41.6)
Empirical evidence provided within source document^a^	704 (40.0)
Literature review	513 (29.2)
Systematic and/or scoping review	290 (16.5)
Assessment of reporting completeness^b^	170 (9.7)
Other type of review	68 (3.9)
Expert consensus	373 (21.2)
Interview	12 (0.7)
Case study	2 (0.1)
Survey	1 (0.06)
Citation(s) provided to other document(s)^a^	582 (33.1)
Citations to existing reporting guidelines	
SPIRIT-PRO	253 (14.4)
CONSORT-PRO	241 (13.7)
CONSORT	141 (8.0)
SPIRIT	42 (2.4)
Other CONSORT extensions	26 (1.5)
Citations to selected key guidance documents^c^	
ICH E6 Good Clinical Practice Guideline	71 (4.0)
International Society for Quality of Life Research (ISOQOL)-recommended PRO reporting standards	14 (7.9)
ICH E9 Statistical Principles for Clinical Trials	8 (0.5)
International Committee of Medical Journal Editors (ICMJE)	7 (0.4)
ICH E3 Structure and Content of Clinical Study Reports	5 (0.3)
Initiative on Methods, Measurement, and Pain Assessment in Clinical Trials (IMMPACT) publication	4 (0.2)
ClinicalTrials.gov guidelines	3 (0.2)
Analgesic, Anesthetic, and Addiction Clinical Trial Translations, Innovations, Opportunities, and Networks (ACTTION) publications	2 (0.1)

*CONSORT* Consolidated Standards of Reporting Trials, *ICH* International Council for Harmonisation of Technical Requirements for Pharmaceuticals for Human Use, *PRO* Patient Reported Outcomes, *SPIRIT* Standard Protocol Items: Recommendations for Interventional Trials

^a^Empirical evidence within the source document and citation provided to other document categorizations were not mutually exclusive, nor are the subcategories within each

^b^Includes any type of literature review that aimed to assess the completeness of reporting in the included articles from either an original review or a secondary analyses of documents included in a prior review

^c^The complete list of citations provided to other documents can be found in the online dataset

Comparison of each of the 1758 recommendations with the initial list of 70 candidate items led to the development of an additional 61 unique candidate reporting items (Table 5). Team discussions produced two additional candidate reporting items, producing a list of 133 candidate reporting items categorized within the ten descriptive categories. One item was excluded by consensus by the Operations Team as the recommendation was consistent with recognized poor methodological practice, yielding 132 candidate reporting items in total (Table 5). The number of candidate items that mapped to each of the ten descriptive categories was variable (range 2–41 items per category), with the largest number of candidate items mapped to the “Outcome data management and analyses” (*n* = 41 items) and the “How: the way the outcome is measured” (*n* = 26 items) categories (Table 5). Most of the recommendations made (*n* = 1611, 91%) could be mapped to a specific candidate reporting item; 153 (9%) were general in nature and were mapped generally to the appropriate category. For example, the recommendation “state how outcome was measured” would be too general in nature to map to a specific candidate item and instead would be mapped to the overall “How: the way the outcome is measured” category. No documents provided an explicit recommendation that refuted or advised against reporting any of the 132 candidate items.

**Table 5:** Total number of documents identified containing a trial outcome reporting recommendation supporting each of the 132 candidate outcome reporting items in total, and number of documents with recommendations made that were specific to protocols, reports, both protocols and reports, and generally for trial documents

Candidate outcome reporting items within ten descriptive categories	Total number of documents (***N***, %)		Number for protocols	Number for reports	Number for protocols and reports	Number generally
**What: description of the outcome (*n*** **= 15 items)**						
Category of “What” in general (recommendation not specific to any candidate item)	36	15	10	17	0	9
State the outcome	84	34	38	26	3	17
Specify the outcome as primary (or secondary)	82	34	30	35	3	14
If a primary outcome, provide a rationale for classifying the outcome as primary	7	3	3	3	1	0
Report if outcome is planned or unplanned^a^	1	0	N/A	1	N/A	0
If a composite outcome, describe all individual components	2	1	0	1	0	1
If a composite outcome, provide citation to methodological paper(s), if applicable^a^	1	0	1	0	0	0
Specify the outcome domain^b^	13	5	5	6	0	2
Provide a rationale for the selected outcome domain^a,b^	2	1	0	1	0	1
Classify the outcome and the outcome domain according to a standard outcome classification system or taxonomy^a,b^	4	2	2	1	0	1
Specify if the outcome is an efficacy or harm outcome (adverse event). If a harm, see CONSORT for harms for specific guidance for trial reports	12	5	10	2	0	0
If outcome is patient-reported, refer to CONSORT-PRO or SPIRIT-PRO for specific guidance, as appropriate^c^	0	0	0	0	0	0
Specify cut-off value for the outcome, if the outcome is continuous but defined and analysed as categorical, and justify cut-off value^a^	5	2	2	1	0	2
Define clinical significance/meaningful change in terms of the outcome (e.g., minimal important difference, responder definition), including what would constitute a good or poor outcome	29	12	16	7	1	5
Justify the criteria used for defining meaningful change including what would constitute a good or poor outcome, such as from an outcome measurement interpretation guideline	5	2	2	2	0	1
Describe underlying basis for determining the criteria used for defining meaningful change^a^	2	1	2	0	0	0
**Why: rationale for selecting the outcome (*n*** **= 10 items)**						
Category of “Why” in general (recommendation not specific to any candidate item)	15	6	10	4	0	1
Explain how the outcome relates to the hypothesis of the study	22	9	7	11	2	2
Explain how the outcome addresses the objective/research question of the study	16	7	10	4	0	2
Explain the mechanism (e.g., pathophysiological, pharmacological, etc.) or theoretical framework/model by which the experimental intervention is expected to cause change in the outcome in the target population	9	4	3	4	0	2
Specify if a relevant core outcome set is publicly available (e.g., via www.comet-initiative.org/), and if so, if the outcome is part of a core outcome set. If applicable, specify which core outcome set the outcome is part of	3	1	1	2	0	0
If a completely new outcome, justify why other outcomes are not appropriate or relevant for use in this trial	1	0	0	0	1	0
If there are other published definitions of the outcome beside the one that was used, explain why the chosen definition was used	0	0	0	0	0	0
Describe why the outcome is relevant to stakeholder groups (e.g., patients, clinicians, funders, etc.)	2	1	0	2	0	0
Report which stakeholders (e.g., patients, clinicians, funders, etc.) are actively involved in outcome selection, as per available guidance for the reporting of patient and public involvement	2	1	1	1	0	0
If applicable, describe discrepancies between the selected outcome and outcomes shown to be of interest to relevant stakeholder groups (e.g., through a core outcome set), and ways to reconcile discrepancies^a^	3	1	0	2	0	1
Provide rationale for the choice of the specific type of outcome (e.g., why a patient-reported outcome instead of a clinician-reported outcome)	7	3	3	3	1	0
**How: the way the outcome is measured (*n*** **= 26 items)**						
Category of “How” in general (recommendation not specific to any candidate item)	59	24	23	28	1	7
Describe the outcome measurement instrument (e.g., questionnaire, laboratory test). If applicable, include instrument scaling and scoring details (e.g., range and direction of scores)	53	22	23	19	0	11
Justify the selection of the outcome measurement instrument^a^	15	6	9	6	0	0
If applicable, specify where outcome measurement instrument materials can be accessed. For trial protocols only: if materials are not publicly available, provide a copy^a^	4	2	3	1	0	0
Specify if more than one language version of the outcome measure instrument used, and if yes, state how the translated versions were developed	3	1	3	0	0	0
If applicable, specify use of outcome measurement instrument in accordance with any user manual, and specify and justify deviations from user manual	10	4	9	1	0	0
If a new or untested outcome measurement instrument, describe an explicit framework (e.g., pathophysiological rationale) and/or supporting clinimetrics to support its use^a^	2	1	0	2	0	0
If assessing multiple outcomes, specify any standardization of order of administration of the outcome measurement instrument(s)	1	0	1	0	0	0
If applicable, specify which outcome measurement instrument(s) is used at each assessment time point^a^	2	1	2	0	0	0
Describe level at which the outcome is measured (i.e., cluster or individual)^a^	2	1	0	2	0	0
Describe any additional resources/materials or processes necessary to perform outcome assessment, when relevant (e.g., language interpreter)	6	2	4	1	0	1
If applicable, specify the recall period for outcome assessment	3	1	3	0	0	0
Describe mode of outcome assessment (e.g., face to face, telephone, electronically)	15	6	8	5	1	1
Justify mode of outcome assessment (e.g., equivalence between different modes of administration)	1	0	1	0	0	0
Describe or provide reference to an empirical study that established validity of the outcome measure instrument for the mode of assessment used in this study^a^	1	0	0	1	0	0
Describe or provide reference to an empirical study that establishes the validity of the outcome measurement instrument in individuals similar to the study sample	39	16	15	19	1	4
If outcome measurement instrument is known to have poor validity in individuals similar to the study sample, described how this discrepancy is accounted for^a^	1	0	0	1	0	0
Describe or provide reference to an empirical study that established validity of the outcome measure instrument in the study setting	35	14	13	19	1	2
Describe or provide reference to an empirical study that established reliability of the outcome measure instrument in individuals similar to the study sample	27	11	8	14	1	4
Describe or provide reference to an empirical study that established reliability of the outcome measure instrument in individuals similar to the study setting	28	11	8	14	1	5
Describe or provide reference to an empirical study that establishes the responsiveness of the outcome measurement instrument in the study sample	3	1	3	0	0	0
Describe level of imprecision of outcome measurement instrument^a^	1	0	0	1	0	0
Describe the feasibility of the outcome measurement instrument in the study sample	0	0	0	0	0	0
Describe the acceptability and burden of the outcome measurement instrument in the study sample	3	1	3	0	0	0
Describe any health risk(s) of the outcome assessment procedure^b^	0	0	0	0	0	0
If applicable, describe any mathematical manipulation of the data necessary to perform during outcome assessment^a^	1	0	0	1	0	0
Specify any monitoring of outcome data during the trial for the purpose of informing the clinical care of individual trial participants, and if applicable, describe how monitoring is managed in a standardized way	2	1	2	0	0	0
**Who: source of information of the outcome (*n*** **= 12 items)**						
Category of “Who” in general (recommendation not specific to any candidate item)	0	0	0	0	0	0
Describe who assesses the outcome (e.g., nurse, parent) in each study group, and if applicable, how many assessors there are	24	10	12	10	0	2
Justify the choice of outcome assessor(s) (e.g., proxy versus healthcare provider)	2	1	2	0	0	0
Describe if there is an endpoint adjudication committee and if so, when the committee will perform the adjudication^a^	4	2	4	0	0	0
Describe any processes to maximize outcome data quality (e.g., duplicate measurements)	17	7	8	9	0	0
Describe any trial-specific training required for outcome assessors to apply the outcome measurement instrument	16	7	9	5	0	2
Describe masking procedure(s) for outcome assessors, outcome data entry personnel, and/or outcome data analysts	20	8	6	10	1	3
Describe if outcome assessor(s) are masked to the intervention assignment	32	13	8	21	0	3
Specified any masking of members of the endpoint adjudication committee to the participant’s intervention group assignment^a^	2	1	1	1	0	0
If applicable, justify why masking was not done, or explain why it was not possible, for outcome assessors, data entry personnel, and/or data analysts^a^	4	2	1	3	0	0
State any strategies undertaken to reduce the potential for unmasking of outcome assessors, data entry personnel, and/or data analysts^a^	4	2	4	0	0	0
If measured, describe success of masking of outcome assessors, outcome data entry personnel, and/or outcome data analysts to intervention assignment^a^	6	2	N/A	5	N/A	1
Specify the name, affiliation, and contact details for the individual(s) responsible for the outcome content to identify the appropriate point of contact for resolution of any outcome-specific inquiries	4	2	3	0	0	1
**Where: assessment location and setting of the outcome (*n*** **= 3 items)**						
Category of “Where” in general (recommendation not specific to any candidate item)	0	0	0	0	0	0
Describe setting of outcome assessment for each study group (e.g., community clinic, academic hospital)	15	6	6	9	0	0
Specify geographic location of outcome assessment for each study group (e.g., list of countries)	10	4	4	6	0	0
Justify suitability of the outcome assessment setting(s) for the study sample (e.g., measuring blood pressure in clinic vs. home)	0	0	0	0	0	0
**When: timing of measurement of the outcome (*n*** **= 2 items)**						
Category of “When” in general (recommendation not specific to any candidate item)	1	0	0	1	0	0
Specify timing and frequency of outcome assessment(s) (e.g., time point for each outcome, time schedule of assessments)	74	30	32	28	3	11
Provided justification of timing and frequency of outcome assessment(s) (e.g., related to pathophysiological evidence for treatment response or complications occurrence and/or pragmatic justification)	9	4	5	4	0	0
**Outcome data management and analyses (*n*** **= 41 items)**						
Category of “Outcome data management and analyses” in general (recommendation not specific to any candidate item)	24	10	14	3	1	6
*Data management and processes*						
Describe outcome data entry, coding, security and storage, including any related processes to promote outcome data quality (e.g., double entry, range checks from outcome data values). Reference to where details of data management procedures can be found, if not included	19	8	16	3	0	0
If applicable, specify who designs the electronic case report form, the name of the data management system, and if it is compliant with jurisdictional regulations^a^	4	2	4	0	0	0
*Analyses*						
Describe analysis metric for the outcome (e.g., change from baseline, final value, time to event)	19	8	14	3	0	2
Describe method of aggregation for the outcome data (e.g., mean, median, proportion)	17	7	10	5	0	2
Described relevant level of precision (e.g., standard deviation) of the outcome data^a^	8	3	2	4	0	2
Describe unit of analysis of the outcome (i.e., cluster or individual)	10	4	2	8	0	0
If applicable, describe any transformations of the outcome data^a^	4	2	2	2	0	0
Provide definition of analysis population relating to protocol non-adherence (e.g., as randomized analysis)	39	16	23	12	1	3
Justify definition of analysis population relating to protocol non-adherence (e.g., as randomized analysis)^a^	1	0	1	0	0	0
Describe specific plans on how to present outcome data (including harms) (e.g., tables, graphs, etc.)^a^	5	2	3	N/A	N/A	2
Describe time period(s) for which the outcome is analysed	29	12	24	3	0	2
If the outcome is assessed at several time points after randomization, state the pre-specified time point of primary interest^a^	7	3	5	2	0	0
Describe statistical/analytical methods and significance test(s) for analysing the outcome data. This should include any analyses undertaken to address risk of type I error, particularly for trials with multiple outcomes and/or measurement time points. Reference to where other details of the statistical analysis plan can be found, if applicable	75	31	41	25	2	7
Justify statistical method(s) for the outcome analyses^a^	5	2	2	3	0	0
State if outcome is part of any interim analyses^a^	4	2	2	2	0	0
If interim analyses of the outcome are performed, describe the method to adjust for this in the final analysis^a^	4	2	3	0	0	1
If applicable, describe methods for additional analyses, such as subgroup analyses and adjusted analyses	27	11	13	11	0	3
Identify statistical software for outcome analysis (e.g., SAS, R)^a^	1	0	0	1	0	0
Describe how the outcome data are assessed for meeting assumptions for the statistical tests selected (e.g., normality, homogeneity of variance, etc.)^a^	7	3	5	1	0	1
Specify alternative statistical methods to be used if the underlying assumptions (e.g., normality) do not hold^a^	2	1	2	0	0	0
Describe how the statistical methods planned to evaluate the outcome are evaluated before implementation (e.g., through the use of simulations)^a^	1	0	0	1	0	0
If applicable, describe any covariates/factors in the statistical model (e.g., adjusted analyses) used for analysing the outcome data	23	9	11	7	1	4
If applicable, justify inclusion and choice of covariates/factors	5	2	2	3	0	0
State and justify the criteria used to exclude any outcome data from the outcome analysis and reporting (e.g., unused data, spurious data)^a^	14	6	12	2	0	0
If applicable, discuss the available power for secondary hypothesis testing for outcomes considered secondary^a^	1	0	1	0	0	0
If intending to report the results of underpowered analyses, state an explicit strategy for their interpretation^a^	2	1	1	N/A	N/A	1
Describe how any unplanned repeat measurements are handled when analysing the outcome data	2	1	0	2	0	0
Specify who analyses the outcome data (e.g., name and affiliation)	10	4	8	1	0	1
*Results*						
Report the number of participants assessed for the outcome^a^	9	4	N/A	5	N/A	4
For each group, specify the number of participants analysed for the outcome^a^	20	8	N/A	19	N/A	1
Describe results for each group, and estimated effect size and its precision (such as 95% confidence interval). For binary outcomes, presentation of both absolute and relative effect sizes is recommended	45	18	N/A	36	N/A	9
Provide the results of planned outcome analyses (regardless of statistical significance)	27	11	N/A	23	N/A	4
Describe results of outcome data at each pre-specified time point^a^	3	1	N/A	3	N/A	0
If a composite outcome, report results of its individual components^a^	3	1	N/A	2	N/A	1
If applicable, separate pre-specified statistical analyses from post-hoc analyses that were not pre-specified^a^	7	3	N/A	6	N/A	1
Report aggregated values of all outcome data (e.g., a table with mean, proportion, etc.) for each group^a^	9	4	N/A	6	N/A	3
Describe results of any other analyses performed, including subgroup analyses and adjusted analyses, distinguishing pre-specified from exploratory^a^	6	2	N/A	6	N/A	0
If the outcome is used to make clinical decisions, provide an effect measure to quantify treatment effects (e.g., number needed to treat)^a^	5	2	N/A	4	N/A	1
If the outcome data is part of a statistical analysis, state where the raw data are accessible (or will be accessible)^a^	8	3	2	6	0	0
Report the statistical code used to complete each outcomes analyses (or where it is/will be accessible)^a^	2	1	1	0	0	1
If someone other than a member in the study group interprets the outcome data, describe the person’s affiliations^a^	3	1	3	0	0	0
**Missing outcome data (*n*** **= 9 items)**						
Category of “Missing outcome data” in general (recommendation not specific to any candidate item)	3	1	2	1	0	0
Describe any plans to minimize missing outcome data	11	5	9	N/A	N/A	2
Describe plans on how reasons for missing outcome data will be recorded^a^	3	1	3	N/A	N/A	0
Describe outcome data collection, assessment process, and analysis for participants who discontinue or deviate from the assigned intervention protocol	14	6	13	1	0	0
Describe methods to calculate missing outcome data rates and assess patterns of missing outcome data^a^	1	0	1	0	0	0
For each group, describe how much outcome data are missing	22	9	N/A	14	N/A	8
For each group, describe any reason(s) for missing outcome data (e.g., missing study visits, lost to follow-up)	17	7	N/A	10	N/A	7
Describe statistical methods to handle missing outcome items or entire assessments (e.g., multiple imputation)	53	22	29	17	2	5
If applicable, describe any analyses conducted to assess the risk of bias posed by missing outcome data (e.g., comparison of baseline characteristics of participants with and without missing outcome data)	4	2	0	3	0	1
Provide justification for methods to handle missing outcome data. This should include: assumptions underlying the missing outcome data mechanism with justification (including analyses performed to support assumptions about the missingness mechanism); and how the assumed missingness mechanism and any relevant features of the outcome data would influence the choice of statistical method(s) to handle missing outcome data including sensitivity analyses	19	8	6	9	0	4
**Interpretation (*n*** **= 11 items)**						
Category of “Interpretation” in general (recommendation not specific to any candidate item)	7	3	1	5	0	1
If there are elements in the clinical trial that would be different in a routine application setting (e.g., patient prompts/reminders, training sessions), discuss what impact the omission of these elements could have on outcomes if the intervention is applied outside the study setting^a^	4	2	0	4	0	0
Report how the outcome results address the trial hypothesis, including the definition of clinically meaningful change, if applicable^a^	2	1	N/A	1	N/A	1
Report how the outcome results addresses the research objective^a^	1	0	N/A	1	N/A	0
Interpret outcome data in relation to clinical outcomes including survival data, where relevant	13	5	N/A	11	N/A	2
Discuss the possibility that the results are caused by type I or type II errors (e.g., multiple outcomes assessed, small sample size)^a^	4	2	N/A	4	N/A	0
Describe other considerations or procedures that could affect the ability to interpret the outcome results	18	7	N/A	13	N/A	5
If applicable, discuss impact of missing outcome data on the interpretation of findings	7	3	N/A	4	N/A	3
If applicable, discuss limitations related to the lack of blinding of outcome assessors, outcome entry personnel, and/or outcome data analysts^a^	3	1	N/A	3	N/A	0
If applicable, discuss any problems with statistical assumptions and/or data distributions for the outcome that could affect the validity of trial results^a^	1	0	N/A	1	N/A	0
If a multi-centre trial, discuss any sources of variability in outcome assessment and the potential impact on trial result(s)^a^	1	0	N/A	1	N/A	0
Interpret potential impact of imprecision on outcome results^a^	3	1	N/A	3	N/A	0
**Modifications (*n*** **= 3 items)**						
Category of “Modifications” in general (recommendation not specific to any candidate item)	1	0	0	0	0	1
Describe any changes to trial outcomes after the trial commenced (e.g., status of primary, definition), with reasons	27	11	1	23	1	2
Described any changes to trial outcomes since the trial was registered, with reasons^a^	2	1	0	2	0	0
Described whether any changes made to the planned analysis of outcomes (including omissions) after the trial commenced, with reasons	15	6	3	5	5	2

*CONSORT* Consolidated Standards of Reporting Trials, *PRO* Patient Reported Outcomes, *SPIRIT* Standard Protocol Items: Recommendations for Interventional Trials. N/A indicates item content was not applicable to trial protocols (e.g., pertained specifically to known trial results) or trial reports (e.g., pertained to trial planning only). Items without footnote a or c are those from the initial list of 70 candidate items

^a^A new item identified from this scoping review (*n* = 61 unique candidate items added from this review in total)

^b^Outcome domain in this context refers to a relatively broad aspect of the effect of illness within which an improvement may occur in response to an intervention; domains may not be directly measurable themselves, so outcomes are selected to assess change within them [43]

^c^A new item generated through Operations Team discussions when the scoping review findings were reviewed for new items

The number of documents containing an outcome reporting recommendation supporting the description of each of the 132 candidate items ranged widely (median 5, range 0–84 documents per item, from a total possible sample of 244 documents; Table 5 and Additional file 5: eFigure 1). Of the 132 candidate reporting items, 104 were applicable to both trial protocols and reports, 24 were not applicable to trial protocols (e.g., pertained specifically to known trial results), and 4 were not applicable to trial reports (e.g., pertained to trial planning only). Comparison with the items and concepts covered in SPIRIT 2013 showed that 78 of the 108 (72%) candidate items relevant to protocols are not currently covered either completely or in part by items in the existing SPIRIT checklist. Comparison with items covered in CONSORT 2010 showed that 106 of the 128 (83%) candidate items relevant to trial reports are not currently covered either completely or in part in the existing CONSORT checklist.

## Discussion

We performed a review of clinical trial outcome-reporting guidance that encompassed all outcome types, disease areas, and populations from a diverse and comprehensive range of sources. Our findings show that existing outcome reporting guidance for clinical trial protocols and trial reports lacks consistency and is spread across a large number of sources that may be challenging for authors to access and implement in research practice. These results suggest that evidence and consensus-based guidance is needed to help authors adequately describe trial outcomes in protocols and reports transparently and completely to help minimize avoidable research waste.

This review provides a comprehensive, evidence-based set of reporting items for authors to consider when preparing trial protocols and reports. The large number of documents included suggest there is much interest in improving outcome reporting in clinical trial documents. Identified outcome reporting items covered diverse concepts that we categorized across ten categories, and the number of items within each category ranged widely. However, authors wishing to use the reporting items identified in this review would face the challenge of trying to describe a large number of reporting concepts into what is typically expensive journal “real estate” (i.e., limited space for competing papers). To date, no published consensus exists on which of these items are essential and constitute best practice to report. For example, it seems unlikely that authors would commonly have the space allowance to provide descriptions of all 41 items within the “Outcome data management and analyses” category, and it is unknown—in the absence of a consensus process—which of these items may be appropriate or necessary to report for any given trial.

Notably, a considerable number of the recommendations we identified are not covered in content or in principle in the existing SPIRIT and CONSORT reporting guidelines [20, 21]. Currently, SPIRIT requires more information on trial outcomes to be reported, and in greater detail, than CONSORT [20, 21]. The results of this review, however, showed that most of the candidate items had a similar number of supporting documents that advocated for their inclusion in protocols and in reports, with a few notable exceptions. For example, 24 documents explicitly supported describing the time period(s) for which the outcome is analysed in trial protocols, but only three suggested including this in trial reports. The exclusion of a clear statement of the planned time period(s) of analyses in trial reports enables the possibility of data analysis “cherry-picking” (e.g., multiple unplanned analyses are performed for multiple measurement time points, with only results for the significant analyses being reported). Consulting other trial documents, such as trial protocols and statistical analyses plans [44], may help mitigate the need for such information in the trial report itself. However, these trial documents may not be publicly available [45] and one must also consider the burden on the knowledge user of needing to consult multiple information sources in an era of transition to online publication methods and free sharing platforms.

In order to identify the minimum set of reporting items it is necessary to include in all clinical trial protocols and reports, respectively, the results of this scoping review were consolidated and presented during the recently held international Delphi survey and expert Consensus Meeting to determine which candidate items should be included or excluded in the SPIRIT-Outcomes and CONSORT-Outcomes extensions and to develop the wording of the final reporting items. This protocol for this process has been described in detail elsewhere [46] and the results are being prepared for publication as part of the extension statements.

### Strengths and limitations

We used a scoping review methodology [32] to map guidance on trial outcome reporting from multiple information sources in an attempt to capture guidance produced and used by relevant stakeholders, including from academic journals, regulatory and government agencies, and ethics review boards [30]. Sensitivity and accuracy may have been reduced by not completing screening and charting in duplicate, although the reviewer training results and periodic data checks by the senior reviewer as well as the fact that all reviewers have graduate-level epidemiological training may have limited this risk. Furthermore, the mapping of every recommendation extracted to each candidate item was verified by the senior reviewer and all of the mapping results presented achieved consensus.

The development of new candidate reporting items followed a planned standardized process of team review and discussion that aimed to minimize item content redundancy and ensure correct interpretation of the extracted recommendations [30]. There may be relevant documents published outside the included date range, and the language restrictions employed yielded a sample of documents that were almost entirely published in English. The international ethics review board websites search represented a convenience sample and therefore may not be representative, for example, of guidance provided by non-English speaking and/or smaller institutions. We were limited to documents that were publicly available or available through our institutional access; in particular, ethics review boards may provide guidance to local investigators that is not publicly available to access. However, using sensitive search methods, saturation was reached such that no new items were identified well prior to the end of document review and charting. Most new items were identified in the initial stages of the review.

Our review focused on the quantity of documents supporting each recommendation and did not formally assess their quality. To help gauge the credibility of gathered recommendations, we categorized the type(s) of underpinning empirical evidence for each recommendation. Indeed, some candidate items were supported by multiple well-recognized sources and had an empirical evidence base or process that underpinned the recommendations as to why this item is recommended to be reported (e.g., from a systematic review or Delphi process). Others were less frequently recommended for reporting or did not provide supporting empirical evidence, but still may have important implications and merit for reporting. For example, a clear recommendation to “identify the outcomes in a trial report as planned (i.e., pre-specified) or unplanned” was found in only one document. However, selective outcome reporting and outcome switching has been well documented in trial reports, is often difficult to detect, and has been shown to impact treatment estimates in meta-analyses [3, 9–11, 14]. The results from the Delphi and consensus processes will help clarify the relative importance and acceptability of the candidate items by an international group of expert stakeholders.

## Conclusions

There is a lack of harmonized guidance to lead authors, reviewers, journal editors, and other stakeholders through the process of ensuring that trial outcomes are completely and transparently described in clinical trial protocols and reports. Existing recommendations are spread across a diverse range of sources and vary in breadth and content. The large number of documents identified, despite limiting our search to the last decade, indicate a substantial interest and need for improving outcome reporting in clinical trial documents. To determine which outcome reporting recommendations constitute best practices for outcome reporting for any clinical trial, a minimum, essential set of reporting items will be identified through evidence and consensus-based methods and ultimately developed into the SPIRIT-Outcomes and CONSORT-Outcomes reporting guidelines.

## Supplementary information

**Additional file 1.** Preferred Reporting Items for Systematic reviews and Meta-Analyses extension for Scoping Reviews (PRISMA-ScR) Checklist.

**Additional file 2.** Electronic database searches.

**Additional file 3.** Grey literature information sources.

**Additional file 4.** Comparison of item content with SPIRIT 2013 and CONSORT 2010 and the number of documents identified containing a trial outcome reporting recommendation supporting each of item, by the reported application of the recommendations to specific outcomes or trial types.

**Additional file 5.** Number of documents containing an outcome reporting recommendation supporting each of the 132 candidate outcome reporting items.

## Data Availability

Project materials and the datasets generated and analysed during the current study are available on the Open Science Framework [39].

## Abbreviations

CONSORT: Consolidated Standards of Reporting Trials
InsPECT: Instrument for reporting Planned Endpoints in Clinical Trials
SPIRIT: Standard Protocol Items: Recommendations for Interventional Trials
