# Supplementary material for: Outcome reporting recommendations for clinical trial protocols and reports: a scoping review
Source: Trials. 2020 Jul 8;21:620. doi: 10.1186/s13063-020-04440-w (PMC7341657; doi:10.1186/s13063-020-04440-w)
Supplement: Supplementary file 2 — Additional file 2. Electronic database searches. [file 13063_2020_4440_MOESM2_ESM.docx]

**Additional file 2.**

**eTable 2. Electronic database searches, performed March 19^th^, 2018.**

| **A. Ovid MEDLINE(R) Epub Ahead of Print, In-Process & Other Non-Indexed Citations, Ovid MEDLINE(R) Daily and Ovid MEDLINE(R) 1946 to Present** | |
| --- | --- |
| **Search term** | **Yield** |
| 1. "outcome and process assessment (health care)"/ or "outcome assessment (health care)"/ or patient outcome assessment/ or critical care outcomes/ or minimal clinically important difference/ or patient reported outcome measures/ or treatment outcome/ or "process assessment (health care)"/ or Endpoint Determination/ | 920893 |
| 2. ((outcome or outcomes or endpoint or endpoints or end point or end points or efficacy variable or efficacy variables or study variable or study variables or dependent variable or dependent variables or efficacy parameter or efficacy parameters or efficacy parametre or efficacy parametres or primary objective or primary objectives or response variable or response variables or effect estimate or effect estimates) adj15(trial or trials)).tw,kf | 75880 |
| 3. 1 or 2 | 971463 |
| 4. peer review, research/ or research design/ or research report/ or writing/ or medical writing/ or publishing/ or open access publishing/ or documentation/ | 153834 |
| 5. (report or reporting or write or writing or written or document or documentation).tw,kf. | 1838670 |
| 6. 4 or 5 | 1964106 |
| 7. (guideline adherence/ or guidelines as topic/ or practice guidelines as topic/ or checklist/) | 157653 |
| 8. (((recommendation or recommendations or guidance or guideline or guidelines or guide line or guide lines or checklist or checklists or check list or check lists or standard or standards or requirement or requirements or instruction or instructions) adj15 (outcome or outcomes or endpoint or endpoints or end point or end points or efficacy variable or efficacy variables or study variable or study variables or dependent variable or dependent variables or efficacy parameter or efficacy parameters or efficacy parametre or efficacy parametres or primary objective or primary objectives or response variable or response variables)).tw,kf) | 52685 |
| 9. 7 or 8 | 204258 |
| 10. clinical trials as topic/ or clinical trials, phase i as topic/ or clinical trials, phase ii as topic/ or clinical trials, phase iii as topic/ or clinical trials, phase iv as topic/ or controlled clinical trials as topic/ or non-randomized controlled trials as topic/ or randomized controlled trials as topic/ or intention to treat analysis/ or pragmatic clinical trials as topic/ or multicenter studies as topic/ or clinical protocols/ | 341070 |
| 11. (trial or trials or trial protocol or trial protocols or study protocol or study protocols).tw,kf | 864710 |
| 12. 10 or 11 | 1041738 |
| 13. 3 and 6 and 9 and 12 | 2938 |
| 14. ((outcome or outcomes or endpoint or endpoints or end point or end points or efficacy variable or efficacy variables or study variable or study variables or dependent variable or dependent variables or efficacy parameter or efficacy parameters or efficacy parametre or efficacy parametres or primary objective or primary objectives or response variable or response variables).ti.) | 270663 |
| 15. ((report or reporting or write or writing or written or document or documentation or recommendation or recommendations or guidance or guideline or guidelines or guide line or guide lines or checklist or checklists or check list or check lists or standard or standards or requirement or requirements or instruction or instructions).ti.) | 684328 |
| 16. ((trial or trials or trial protocol or trial protocols or study protocol or study protocols).ti.) | 242328 |
| 17. 14 and 15 and 16 | 578 |
| 18. ((statement or statements or reporting or items or item or standard or standards or extension or extensions or guideline or guidelines).ti.) | 181146 |
| 19. ((CONSORT or Consolidated Standards of Reporting Trials or SPIRIT or Standard Protocol Items: Recommendations for Interventional Trials).ti.) | 2372 |
| 20. 18 and 19 | 328 |
| 21. 13 or 17 or 20 | 3688 |
| 22. remove duplicates from 21 | **3682** |

| **B. EBM Reviews - Cochrane Methodology Register 3rd Quarter 2012** | |
| --- | --- |
| **Search term** | **Yield** |
| 1. ((outcome or outcomes or endpoint or endpoints or end point or end points or efficacy variable or efficacy variables or study variable or study variables or dependent variable or dependent variables or efficacy parameter or efficacy parameters or efficacy parametre or efficacy parametres or primary objective or primary objectives or response variable or response variables or effect estimate or effect estimates) adj15(trial or trials)).tw,kf | 1446 |
| 2. (report or reporting or write or writing or written or document or documentation).tw,kf. | 3209 |
| 3. (((recommendation or recommendations or guidance or guideline or guidelines or guide line or guide lines or checklist or checklists or check list or check lists or standard or standards or requirement or requirements or instruction or instructions) adj15 (outcome or outcomes or endpoint or endpoints or end point or end points or efficacy variable or efficacy variables or study variable or study variables or dependent variable or dependent variables or efficacy parameter or efficacy parameters or efficacy parametre or efficacy parametres or primary objective or primary objectives or response variable or response variables)).tw,kf) | 300 |
| 4. (trial or trials or trial protocol or trial protocols or study protocol or study protocols).tw,kf | 8399 |
| 5. 1 and 2 and 3 and 4 | 66 |
| 6. ((outcome or outcomes or endpoint or endpoints or end point or end points or efficacy variable or efficacy variables or study variable or study variables or dependent variable or dependent variables or efficacy parameter or efficacy parameters or efficacy parametre or efficacy parametres or primary objective or primary objectives or response variable or response variables).ti.) | 682 |
| 7. ((report or reporting or write or writing or written or document or documentation or recommendation or recommendations or guidance or guideline or guidelines or guide line or guide lines or checklist or checklists or check list or check lists or standard or standards or requirement or requirements or instruction or instructions).ti.) | 1496 |
| 8. ((trial or trials or trial protocol or trial protocols or study protocol or study protocols).ti.) | 4955 |
| 9. 6 and 7 and 8 | 47 |
| 10. ((statement or statements or reporting or items or item or standard or standards or extension or extensions or guideline or guidelines).ti.) | 1195 |
| 11. ((CONSORT or Consolidated Standards of Reporting Trials or SPIRIT or Standard Protocol Items: Recommendations for Interventional Trials).ti.) | 107 |
| 12. 10 and 11 | 95 |
| 13. 5 or 9 or 12 | 197 |
| 14. remove duplicates from 13 | **197** |
