# Supplementary material for: Outcome reporting recommendations for clinical trial protocols and reports: a scoping review
Source: Trials. 2020 Jul 8;21:620. doi: 10.1186/s13063-020-04440-w (PMC7341657; doi:10.1186/s13063-020-04440-w)
Supplement: Supplementary file 3 — Additional file 3. Grey literature information sources. [file 13063_2020_4440_MOESM3_ESM.docx]

**Additional file 3.**

**eTable 3. Grey literature information sources.**

| **A. Google search strategy** | |
| --- | --- |
| 1. trial outcome guidance 2. trial outcome guidelines 3. trial outcome recommendations 4. trial outcome checklist 5. trial endpoint guidance 6. trial endpoint guidelines 7. trial endpoint recommendations 8. trial endpoint checklist 9. trial efficacy variable guidance 10. trial efficacy variable guidelines 11. trial efficacy variable recommendations 12. trial efficacy variable checklist 13. trial dependent variable guidance 14. trial dependent variable guidelines 15. trial dependent variable recommendations 16. trial dependent variable checklist 17. trial efficacy parameters guidance 18. trial efficacy parameters guidelines 19. trial efficacy parameters recommendations 20. trial efficacy parameters checklist | 1. trial protocol outcome guidance 2. trial protocol outcome guidelines 3. trial protocol outcome recommendations 4. trial protocol outcome checklist 5. trial protocol endpoint guidance 6. trial protocol endpoint guidelines 7. trial protocol endpoint recommendations 8. trial protocol endpoint checklist 9. trial protocol efficacy variable guidance 10. trial protocol efficacy variable guidelines 11. trial protocol efficacy variable recommendations 12. trial protocol efficacy variable checklist 13. trial protocol dependent variable guidance 14. trial protocol dependent variable guidelines 15. trial protocol dependent variable recommendations 16. trial protocol dependent variable checklist 17. trial protocol efficacy parameters guidance 18. trial protocol efficacy parameters guidelines 19. trial protocol efficacy parameters recommendations 20. trial protocol efficacy parameters checklist |

| **B. Targeted website search strategy** (n=41 websites screened after de-duplication of sites identified) |
| --- |
| 1. EQUATOR network (<http://www.equator-network.org/>), including all reporting guideline extensions for SPIRIT and CONSORT 2. World Health Organization (<http://www.who.int/en/>) 3. US Food and Drug Administration (<https://www.fda.gov/>) 4. Health Canada (<https://www.canada.ca/en/health-canada.html>) 5. European Medicines Agency (<http://www.ema.europa.eu/ema/>) 6. National Institutes of Health (NIH) (<https://www.nih.gov/>) 7. Agency for Healthcare Research and Quality (<https://www.ahrq.gov/>) 8. COMET Initiative (<http://www.comet-initiative.org/>) 9. ClinicalTrials.gov (<https://clinicaltrials.gov/>)   *Relevant websites identified from the Canadian Agency for Drugs and Technologies in Health (CADTH) tool^1^*   1. WHO International Clinical Trials Registry Platform search portal (ICTRP) (http://apps.who.int/trialsearch/) 2. WHO Regional Office for Europe Health Evidence Network (http://www.euro.who.int/en/data-and-evidence/evidence-informed-policy-making/publications/by-keyword) 3. Joanna Brigg Institute EBP Database (http://connect.jbiconnectplus.org/Search.aspx) 4. Wiley InterScience Cochrane Library (http://onlinelibrary.wiley.com/o/cochrane/cochrane_search_fs.html) 5. BioMed Central: ISRCTN Registry (http://www.isrctn.com/) 6. Thomson CenterWatch. CenterWatch Clinical Trials Listing Service (http://www.centerwatch.com/clinical-trials/listings/)   *Recommended by colleagues through the solicitation of expert colleagues search*   1. ISPOR – Health Economics and Outcomes Research (https://www.ispor.org/workpaper/practices_index.asp) 2. National Cancer Institute - Division of Cancer Control & Poopulation Sciences : Healthcare Delivery Research Program - Patient-Reported Outcomes version of the Common Terminology Criteria for Adverse Events (PRO-CTCAE™) (https://healthcaredelivery.cancer.gov/pro-ctcae/) 3. UNC Lineberger - Cancer Outcomes Research Program - Pediatric PRO-CTCAE design and validation (https://unclineberger.org/outcomes/research/pediatric-pro-ctcae) 4. AllTrials (http://www.alltrials.net/) 5. Australian New Zealand Clinical Trial Registry (ANZCTR) (http://www.anzctr.org.au/) 6. Australian Clinical Trials (www.australianclinicaltrials.gov.au) 7. Cuban Public Registry of Clinical Trials (RPCEC) (http://registroclinico.sld.cu/en/home) 8. Brazilian Clinical Trials Registry (ReBec) (http://www.ensaiosclinicos.gov.br/) 9. Peruvian Clinical Trial Registry (REPEC) (http://www.ensayosclinicos-repec.ins.gob.pe/en/) 10. Chinese Clinical Trial Registry (ChiCTR) (http://www.chictr.org.cn/) 11. Clinical Research Information Service (CRiS), Republic of Korea (http://cris.nih.go.kr/cris/en/use_guide/cris_introduce.jsp) 12. Clinical Trials Registry - India (CTRI) (http://ctri.nic.in/) 13. Sri Lanka Clinical Trials Registry (SLCTR) (http://www.slctr.lk/) 14. Japan Primary Registries Network (JPRN) (https://rctportal.niph.go.jp/en/) 15. UMIN Clinical Trials Registry (UMIN-CTR) (http://www.umin.ac.jp/ctr/) 16. Japan Pharmaceutical Information Center (http://www.japic.or.jp/) 17. Center for Clinical Trials, Japan Medical Association (http://www.jmacct.med.or.jp/en/) 18. Thai Clinical Trials Registry (TCTR) (http://www.clinicaltrials.in.th/) 19. Iranian Registry of Clinical Trials (IRCT) (http://www.irct.ir/) 20. EU Clinical Trials Register (EU-CTR) (https://www.clinicaltrialsregister.eu/) 21. German Clinical Trials Register (DRKS) (http://www.germanctr.de/) 22. ISRCTN (http://www.isrctn.org/) 23. The Netherlands National Trial Register (NTR) (http://www.trialregister.nl/) 24. Pan African Clinical Trial Registry (PACTR) (http://www.pactr.org/) 25. The SONG Handbook (http://songinitiative.org/reports-and-publications/) 26. PROlearn ([www.birmingham.ac.uk/prolearn](http://www.birmingham.ac.uk/prolearn))   *Recommended by colleagues that were duplicates with other sources*   1. World Health Organization - International Clinical Trials Registry Platform - Search Portal (http://apps.who.int/trialsearch/) 2. EQUATOR Network (http://www.equator-network.org/) 3. National Institutes of Health (https://www.nih.gov/) 4. COMET Initiative (http://www.comet-initiative.org/) 5. NIH - US National Library of Science - ClinicalTrials.gov (https://clinicaltrials.gov/) |

| **C. Ethics review boards search methods^a^** | | |
| --- | --- | --- |
| **Country** | **Universities** | **Hospitals** |
| Australia | University of Melbourne  Australian National University  University of Sydney  University of Queensland  University of New South Wales | Royal Brisbane & Women's Hospital  Westmead Hospital  Princess Alexandra Hospital  Fiona Stanley Hospital  Gold Coast University Hospital |
| Canada | University of Toronto  University of British Columbia  McGill University  University of Alberta  University of Montreal | University Health Network  Hospital for Sick Children  McGill University Health Centre  Hamilton Health Sciences  British Columbia Provincial Health Services Authority |
| United Kingdom | University of Oxford  University of Cambridge  Imperial College London  University College London  University of Edinburgh | Imperial College Healthcare NHS Trust  King’s College Hospital NHS Foundation Trust  Kingston Hospital NHS Trust  University College London Hospitals NHS Foundation Trust  Newham University Hospital NHS Trust |
| United States of America | Harvard University  Massachusetts Institute of Technology  Stanford University  University of California Berkeley  Princeton University | Mayo Clinic  Cleveland Clinic  Johns Hopkins Hospital  Massachusetts General Hospital  University of California San Francisco Medical Centre |

^a^Limited to five major universities and hospitals each in four English-speaking countries for feasibility.

^b^The research universities were identified from the Times Higher Education World University Rankings^34^. Available national ranking systems from each country were used to identify major research hospitals for inclusion; proxy rankings such as highest number of patient admissions were used as needed. Sources used were as follows: Australia, based on number of patient admissions for 2015-2016^3^, which was the most recent data available; Canada, Canada’s Top 40 Research Hospitals^4^; United Kingdom, Dr. Foster Hospital Guide 2011^5^, with five selected from the seven hospitals listed that scored below expected in mortality on Hospital Standardized Mortality Ratio (HSMR), Summary Hospital-level Mortality Indicator (SHMI) and death in low-risk conditions; as these seven hospitals were not individually ranked, the five were selected based on largest number of beds; USA, Best Hospitals U.S. News and World Report Rankings^6^.

| **D. eTable 3 References** |
| --- |
| 1. CADTH Information Services. Grey Matters: a practical tool for searching health-related grey literature 2015 [updated November 2015. Available from: <https://www.cadth.ca/resources/finding-evidence/grey-matters> accessed February 14 2018.  2. THE (Times Higher Education) World University Rankings - Best Universities in Canada 2018 2018 [updated September 5 2017. Available from: <https://www.timeshighereducation.com/student/best-universities/best-universities-canada#survey-answer> accessed February 14 2018.  3. Australian Government Institute of Health and Welfare. Patient admissions [Available from: <https://www.myhospitals.gov.au/about-the-data/download-data> accessed February 14 2018.  4. RE$EARCH Infosource Inc. Canada's Top 40 Research Hospitals 2017 [updated November 16 2017. Available from: <https://www.researchinfosource.com/pdf/CIL2017-Top%2040%20Hospital%20List.pdf> accessed February 14 2018.  5. NHS Choices. Guide rates best and worst hospitals in 2011 2011 [updated November 28 2011. Available from: <https://www.nhs.uk/news/medical-practice/guide-rates-best-and-worst-hospitals-in-2011/> accessed February 14 2018.  6. Comarow A, Harder B. U.S. News and World Report - 2017-18 Best Hospitals Honor Roll and Overview 2017 [updated August 8 2017. Available from: <https://health.usnews.com/health-care/best-hospitals/articles/best-hospitals-honor-roll-and-overview> accessed February 14 2018. |
