# Supplementary material for: Outcome reporting recommendations for clinical trial protocols and reports: a scoping review
Source: Trials. 2020 Jul 8;21:620. doi: 10.1186/s13063-020-04440-w (PMC7341657; doi:10.1186/s13063-020-04440-w)
Supplement: Supplementary file 5 — Additional file 5. Number of documents containing an outcome reporting recommendation supporting each of the 132 candidate outcome reporting items. [file 13063_2020_4440_MOESM5_ESM.pdf]

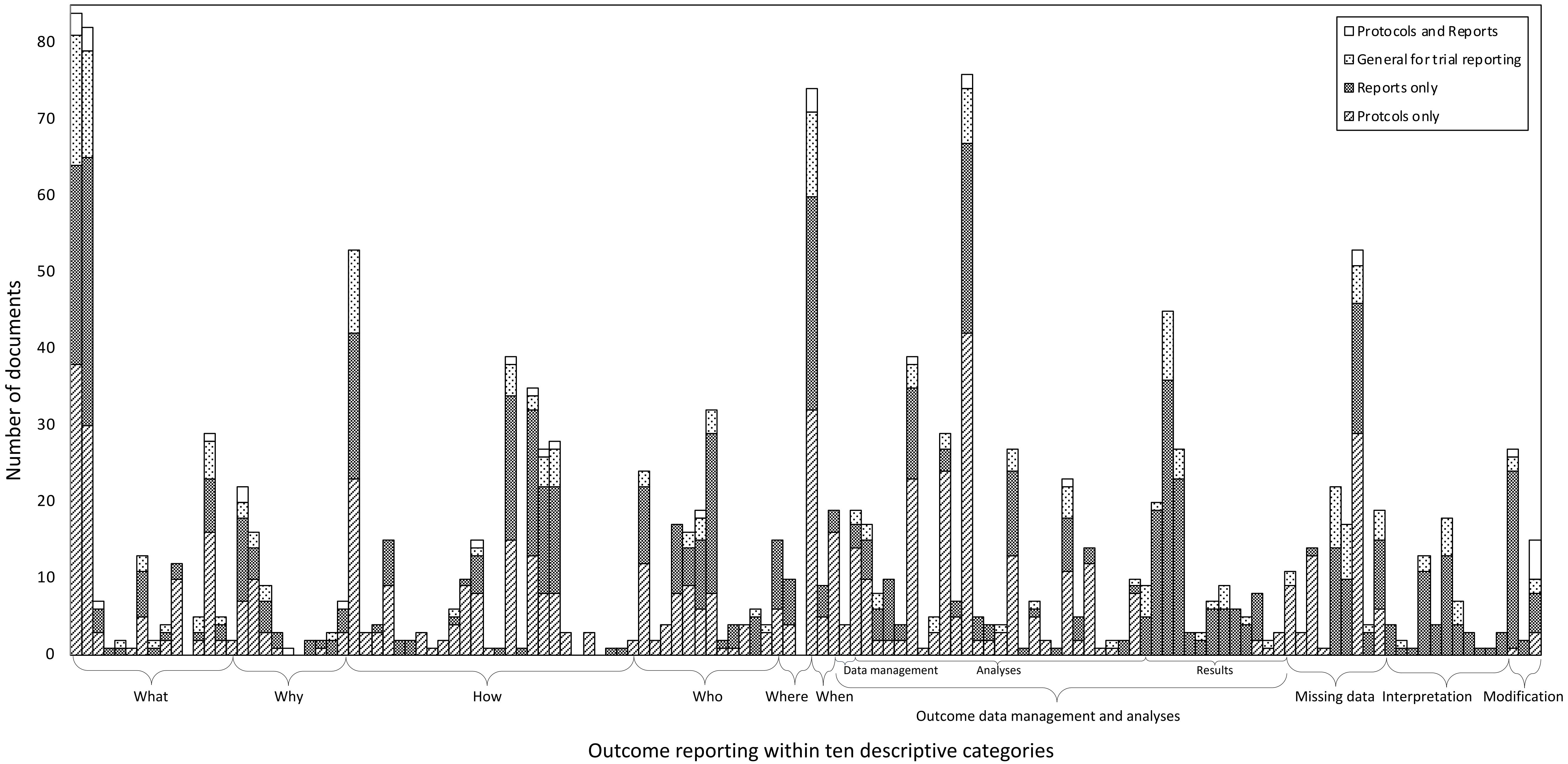

Additional file 5. eFigure 1. Number of documents containing an outcome reporting recommendation supporting each of the 132 candidate outcome reporting items. Bars correspond with ordered items listed in Table 4
